# Analytical modification of EDFM for transient flow in tight rocks

**DOI:** 10.1038/s41598-022-26536-w

**Published:** 2022-12-20

**Authors:** Olufemi Olorode, Harun Rashid

**Affiliations:** Petroleum Engineering, Lousiana State University, 3207 Patrick F. Taylor Hall, Baton Rouge, LA 70803 USA

**Keywords:** Crude oil, Natural gas, Petrol

## Abstract

The commercial development of unconventional resources with multiply fractured horizontal wells has been in the spotlight over the last ten years because of the significant contribution of unconventional oil and gas (UOG) reservoirs to the total US oil and gas production. UOG reservoirs contain multiscale fractures with heterogeneous properties, so the focus has been on efficient and accurate models that can account for these fractures individually. One of such models is the embedded discrete fracture model (EDFM), which has been applied to various types of fractured reservoirs. This work shows that the application of EDFM in fractured tight rocks yields significant errors because it cannot account for the expected transient flow between the matrix and fractures. To address the limitation when EDFM is used in tight rocks with structured Cartesian grids, we modified the matrix/fracture non-neighboring connection (NNC) flux in EDFM by multiplying it with a transient factor. We obtained this factor as in the transient matrix/fracture transfer term for dual-continuum models and implemented it in in our open-source shale simulator. We simulated a single vertical fracture in the middle of a tight reservoir with and without this EDFM modification and show the importance of the proposed modification. We also simulated cyclic gas enhanced oil recovery (CGEOR) in a fractured Bakken shale oil well and analyzed the model results using standard rate-transient analysis plots to evaluate the significance of the proposed modification. The results show that the standard EDFM underestimates oil and gas production by up to 73% at early times. This work presents the first analytical modification of EDFM to account for the nonlinear pressure drop expected near fracture surfaces. Comparing the modified and standard EDFM model results to a reference solution shows that the modified EDFM matches it. In contrast, the standard EDFM cannot match the reference solution when we use structured Cartesian grids with linear spacing. Additionally, by timing the simulation of a representative Bakken shale oil reservoir with 256 fractures, we show that the analytical modification proposed is only 1.5% slower than the standard EDFM.

## Introduction

The development of unconventional oil and gas (UOG) resources has contributed at least 50% of the total US oil and gas production over the last seven years. Despite the low oil and gas prices in 2020, the US 2021 annual EIA outlook data shows that shale oil and gas production contributed 65% and 78% of US oil and gas production, respectively. The increased role of UOG reservoirs in meeting the US energy demand has led to a corresponding increase in research centered on modeling production from fractured tight rocks. Considering the significant contribution of these fractures to flow in these tight rocks, several researchers have developed different mathematical models to simulate flow in fractured rocks. Depending on whether the fractures are modeled individually, these models could be classified as effective medium, discrete, or hybrid models.

As explained in Olorode et al.^1^, the effective models represent the fractured reservoir as an effective medium with homogenized or average properties. These include the dual-porosity^2^, dual-permeability^3^, and multi-continuum models^4^. Unlike the effective medium models, discrete models individually account for each fracture in fractured reservoirs. These include the discrete fracture model (DFM)^5,6^, embedded discrete fracture model (EDFM)^7^, and projection-based embedded discrete fracture model (pEDFM)^8^. Hybrid models essentially combine concepts from the effective medium and discrete fracture models. They include the multiple sub-region (MSR) model^9^ and multi-level DFMs^10,11^. The dual continuum and EDFM models are the most commonly used fracture models^12^.

**Figure 1:**
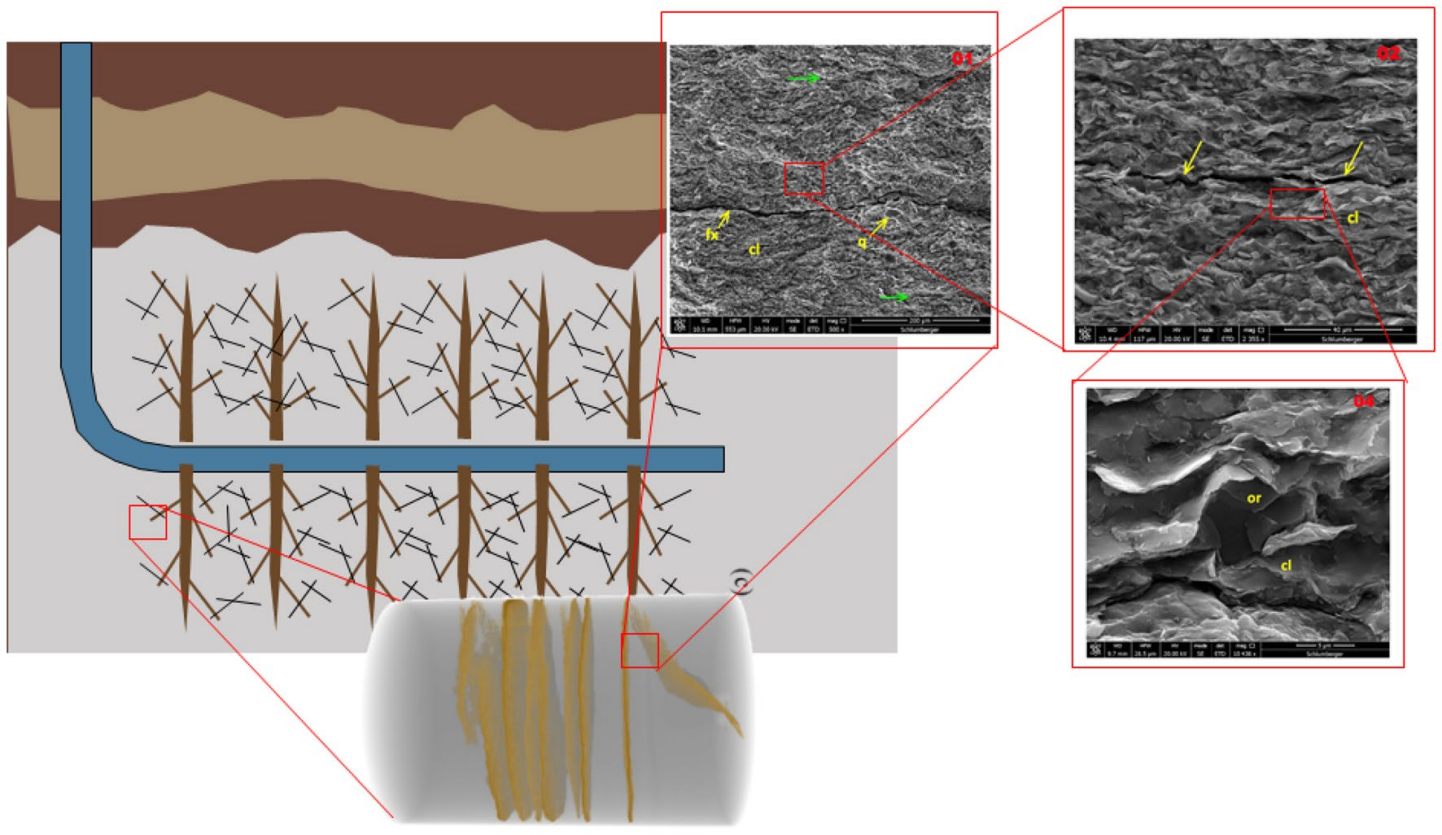
This sketch illustrates the presence of fractures at multiple scales in organic-rich source rocks. The man-made hydraulic fractures are on the order of millimeters and tens/hundreds of meters in aperture and length, respectively, while the micro-cracks are on the order of micrometers in length. The natural fractures are at an intermediate scale between these two and could be on the order of meters in length.

The dual continuum models were developed based on the assumption of a dense fracture distribution with minimal variation in fracture properties, such as aperture, length, and orientation. Additionally, the original dual-porosity model of Warren and Root^2^ is based on a pseudo-steady state (PSS) assumption in the matrix/fracture transfer term. However, shales are known to contain multiscale fractures, as shown in Fig. 1. This, coupled with the heterogeneity expected in the fracture properties, makes the applicability of dual-continuum models in UOG reservoirs questionable. To address the limitation that the PSS transfer term is only applicable at late times, Zimmerman et al.^13^ used the Vermeulen^14^ approach to derive a transient matrix/fracture transfer term that is accurate at early and late times. This transient matrix/fracture transfer term can be rearranged as in Azom and Javadpour^15^ and Olorode et al.^16^, so that it becomes a product of a transient factor and the PSS transfer term.

In contrast to the dual continuum models, the discrete fracture model accounts for the properties and orientation of each fracture in the domain individually. Its limitation is that it is very computationally expensive because the matrix must conform to the geometry and orientation of all the natural fractures in the reservoir. EDFM was developed to address this limitation by meshing the matrix independently of the fractures. For three-dimensional (3D) systems, the matrix is meshed in 3D, whereas the fractures are meshed in 2D. To account for the expected flow between each fracture and its host matrix, EDFM computes a non-neighboring connection (NNC) flux term. This pseudo-steady state NNC flux term is linear in pressure and similar to the PSS transfer term in the dual permeability model.

Although EDFM has been applied to model naturally fractured reservoirs, the assumption of a linear pressure dependence in the matrix/fracture transfer term could lead to significant errors when the matrix permeability is very low^17,18^. In Rao et al.^17^, the authors presented the use of local grid refinements to account for the expected transient matrix/fracture flow. The limitation of this approach is that the total number of cells in the domain will be too large when modeling actual shale plays with thousands of natural fractures, making it computationally inefficient. On the other hand, Rao et al.^18^ proposed using boundary integral equations for transient flow to account for the matrix/fracture flow term in tight rocks. This work presents an analytical modification of the matrix/fracture NNC flux term in EDFM to address the computational inefficiencies of the previous approaches. We show that by multiplying the standard EDFM matrix/fracture term by a transient factor (as in dual continuum models), we obtain a transient NNC term that is applicable at all times (early and late times). The analysis of the results of the simple and elegant implementation of this modification in the MATLAB reservoir simulation toolbox (MRST)^19^ shows that it is both fast and accurate.

This paper starts with an overview of natural fracture modeling with EDFM. We discuss dual continuum modeling with the pseudo-steady state and transient matrix/fracture transfer terms. Next, we present some simulation results that demonstrate the limitation of EDFM at early times. We then modify EDFM to account for the expected transient matrix/fracture transfer in tight rocks. We compare this model to a high-resolution, fully dimensional model with a geometrically spaced mesh to validate it. We also present the results of some mesh sensitivity studies based on the standard and modified EDFM models. Finally, we apply the proposed EDFM modification to cyclic gas enhanced oil recovery (EOR) in a representative naturally fractured Bakken shale-oil reservoir. The results from the case with and without the proposed modification are compared using standard rate-transient analysis plots to show the significance of the analytical EDFM modification proposed.

## Natural fracture modeling with EDFM

EDFM uses the concept of non-neighboring connections (NNCs) to couple the flow of fluids in a fracture cell to that of its host (or embedding) matrix cell. The coupling occurs by adding a $q_i^{nnc}$ term to the semi-discrete form of the governing equation for compositional simulation which is discussed in Appendix A, as follows:

1 $$\begin{aligned} \begin{aligned} \frac{1}{\Delta t} \left[ \left( \phi \rho ^l S^l X^l_i+\phi \rho ^v S^v X^g_i\right) ^{n+1} - \left( \phi \rho ^l S^l X^l_i+\phi \rho ^v S^v X^g_i\right) ^{n}\right] + \\ \quad \nabla \cdot (\rho ^l X^l_i \vec {v}^l+\rho ^v X^g_i \vec {v}^v) -(\rho ^l X^l_i q^l+\rho ^v X^g_i q^v)/V + q_i^{nnc} = R_i, \end{aligned} \end{aligned}$$

where $\phi$, $\rho ^\alpha$, $S^\alpha$, and $q^\alpha$ represent the matrix porosity, mass density, saturation, and volumetric withdrawal/injection rate of phase $\alpha$, respectively. In this equation, phase $\alpha$ corresponds to the liquid (*l*) and vapor (*v*) phases, whereas *V*, $\Delta t$, and $R_i$ represent the grid-block volume, time-step size, and residual of cell *i*, respectively. The divergence operator is written as $\nabla \cdot$, and the superscripts *n* and $n+1$ represent the previous and current time steps, respectively. The symbols $X^l_i$ and $X^g_i$ represent the mass fractions of component *i* in the liquid and vapor phases, while $\vec {v_l}$ and $\vec {v_v}$ are the Darcy velocities for the liquid and vapor hydrocarbon phases, respectively. The symbol $q_i^{nnc}$ is the mass rate of component *i* that is exchanged through the NNC (in units of mass per time). It is given as:

2 $$\begin{aligned} q_i^{nnc} = \frac{1}{V}\sum _{m=1}^{N_{nnc}} A_m^{nnc}\sum _{\alpha =1}^{n_p}\frac{k_m^{nnc} k_{r\alpha }}{\mu ^\alpha }\rho ^\alpha X^\alpha _i\left( \frac{\Phi - \Phi _m^{nnc}}{d_m^{nnc}}\right) , \end{aligned}$$

where subscript *m* is an index from 1 to the total number of non-neighboring connections for each cell ($N_{nnc}$). The flow potentials, $\Phi$ of a cell and its non-neighboring cell are written as $(p^\alpha -\rho ^\alpha g z)$ and $(p^\alpha -\rho ^\alpha g z)^{nnc}$, respectively. The symbols $\mu ^\alpha$ and $k_{r\alpha }$ represent the viscosity of phase $\alpha$ and the relative permeability of the rock to phase $\alpha$, respectively. In single-phase simulations, the relative permeability and phase saturation are one. However, in the multiphase simulations performed, the relative permeability and capillary pressures were obtained from simple polynomial functions of the corresponding saturation, as explained in Sections 8.1.3 and 8.1.4 of Lie^19^. To ensure a reasonable comparison of the standard EDFM to the proposed modified version, we use the same relative permeability and capillary pressure parameters in the multiphase simulations presented in Section 7.

To determine the transmissibility factor ($T^{nnc}$) between any pair of cells that are connected via non-neighboring connections, we need to estimate the area ($A^{nnc}$), permeability ($k^{nnc}$), and distance ($d^{nnc}$) of the non-neighboring connections. This transmissibility factor is given as:

3 $$\begin{aligned} T^{nnc}=\frac{k^{nnc}A^{nnc}}{d^{nnc}}. \end{aligned}$$

**Figure 2:**
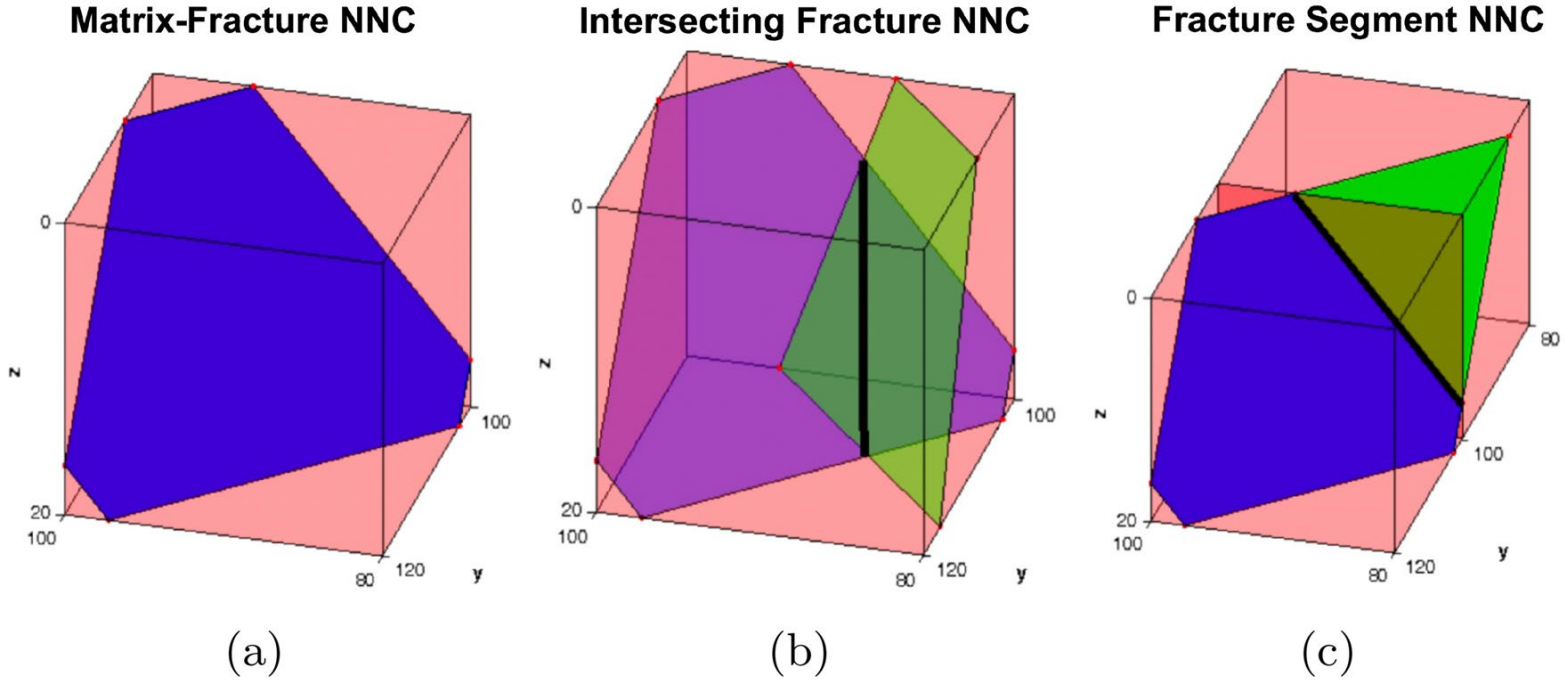
This figure from Moinfar et al.^20^ illustrates (**a**) the NNC between a matrix and a fracture cell, (**b**) two fracture cells that belong to different fracture planes, and (c) two fracture cells that are part of the same fracture plane.

The equations to estimate $A^{nnc}$, $k^{nnc}$, and $d^{nnc}$ are different for different types of NNC. Moinfar et al.^20^ provides more details on these equations and the expressions for the three types of NNCs in EDFM, which are shown in Fig. 2. Of these three, this work focuses on the matrix/fracture NNCs because this is where we expect significant transient flow or nonlinear pressure drop in tight rocks. It is worth noting that the $q_i^{nnc}$ term in Eq. (2) is a linear function of the difference in the flow potential of the matrix cell and that of the fracture cell in matrix/fracture NNCs. This pseudo-steady-state flux assumption in the standard EDFM model is also used in the coupling term of the dual-porosity model of Warren and Root^2^ and is the fundamental reason that EDFM is unable to match the reference solutions at early times, where the flow is transient. The next section introduces the dual-continuum model, and shows the similarity between the EDFM $q_i^{nnc}$ term and the coupling term in dual-continuum models.

## Dual-continuum modeling

Dual-continuum modeling is an effective medium approach that involves treating the matrix and fractures as separate continua. Depending on whether the governing equations are solved in the matrix continuum, this model can either be classified as a dual-porosity model or a dual-permeability model. The dual porosity model solves the governing mass balance equation for the fracture continuum, assumes the pressure in the matrix continuum is constant, and models the transfer of fluids from the matrix to the fracture using the matrix-fracture coupling term. The dual permeability model extends the dual porosity model further, by solving the governing equation in both the matrix and fracture continua, in addition to having the matrix-fracture coupling term.

Considering the striking similarity of the matrix/fracture NNC flux in the EDFM to the pseudo-steady state (PSS) matrix/fracture transfer term in the dual-porosity model^2^, this section begins with a discussion of the dual-porosity model. We discuss the limitation of this model at early times and present the transient matrix/fracture transfer from Zimmerman et al (1993)^13^.

### Pseudo-steady state matrix/fracture transfer in the dual-porosity model

The governing equation for the flow in the fracture continuum in a dual-porosity model is essentially the same as in Eq. (1) but with the $q_i^{nnc}$ term replaced by a coupling term, $Q_i$, which is defined as:

4 $$\begin{aligned} Q_i = \frac{1}{V}\sum _{i=1}^{N_f} A_i\sum _{\alpha =1}^{n_p}\frac{k_m k_{r\alpha }}{\mu ^\alpha }\rho ^\alpha X^\alpha _i\left( \frac{\Phi _f - \Phi _m}{d_i}\right) . \end{aligned}$$

Here, *V* is the volume of the matrix grid block, $A_i$ is the area of each matrix-fracture interface *i*, $d_i$ is the distance between the center of the matrix block and each surface *i*, and $\Phi _f - \Phi _m$ is the potential difference between the fracture and matrix. From Kazemi et al.^21^, the pseudo-steady state shape factor ($\sigma _P$) can be estimated for any matrix block geometry as follows:

5 $$\begin{aligned} \sigma _P = \frac{1}{V}\sum _{i=1}^{N_f} \frac{A_i}{d_i}. \end{aligned}$$

Substituting this into Eq. (4) yields:

6 $$\begin{aligned} Q_i = \sigma _P \sum _{\alpha =1}^{n_p}\frac{k_m k_{r\alpha }}{\mu ^\alpha }\rho ^\alpha X^\alpha _i\left( \Phi _f - \Phi _m\right) . \end{aligned}$$

### Transient matrix/fracture transfer in the dual-porosity model

Zimmerman et al.^13^ showed that the pseudo-steady state assumption in the derivation of $\sigma _P$ is valid at late times but not at early times. They used the Vermeulen^14^ approach to obtain a transient factor ($T_f$), which can be multiplied with the pseudo-steady state shape factor to obtain the transient shape factor ($\sigma _T$) as follows:

7 $$\begin{aligned} \sigma _T = \sigma _P \, T_f, \end{aligned}$$

where the transient factor can be written as:

8 $$\begin{aligned} T_f = \frac{2 \Phi _i - \left( \Phi _m + \Phi _f\right) }{2\left( \Phi _i-\Phi _m\right) }. \end{aligned}$$

In this equation, $\Phi _i$ represents the flow potential at initial conditions. Zimmerman et al.^13^ and Azom and Javadpour^15^ showed that the multiplication of the pseudo-steady state transient factor by the transient factor yields a transient factor that is applicable at all (early and late) times. Considering the striking similarity between the pseudo-steady state matrix/fracture coupling term and the matrix/fracture NNC flux in EDFM, the next section shows the limitation of the EDFM in capturing the transient flow expected in fractured tight rocks.

## Limitation of EDFM at early times

**Figure 3:**
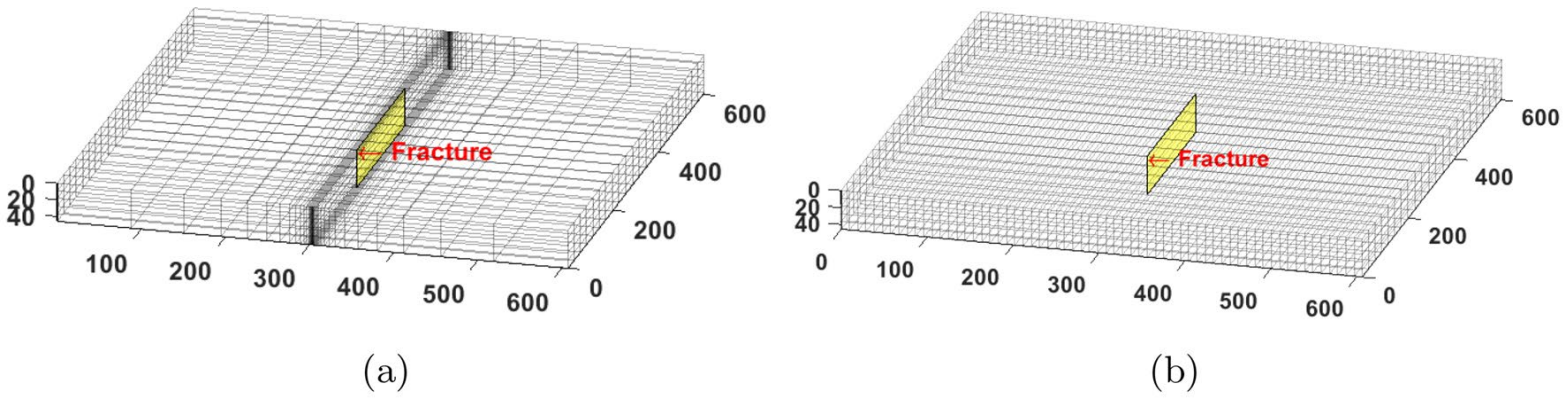
The left image shows geometrically spaced gridding, whereas the right image shows a structured Cartesian mesh.

To show the limitation of the standard EDFM in ultra-low matrix permeability rocks, we simulate three cases with a single vertical fracture in the middle of the reservoir domain. In the first case, we use a fully dimensional model in a geometrically spaced grid. This approach is referred to as the “explicit” model because the fracture segments are modeled individually as 3D cells with a thickness equal to the fracture aperture. The mesh is designed to get larger with increasing distance from the fracture surface, as shown in Fig. 3a. In the second case, we use the same mesh as the explicit one, but the smallest mesh is slightly wider than the fracture aperture. EDFM is then used in the smallest mesh. Finally, the third case models EDFM in the structured mesh shown in Fig. 3b.

**Figure 4:**
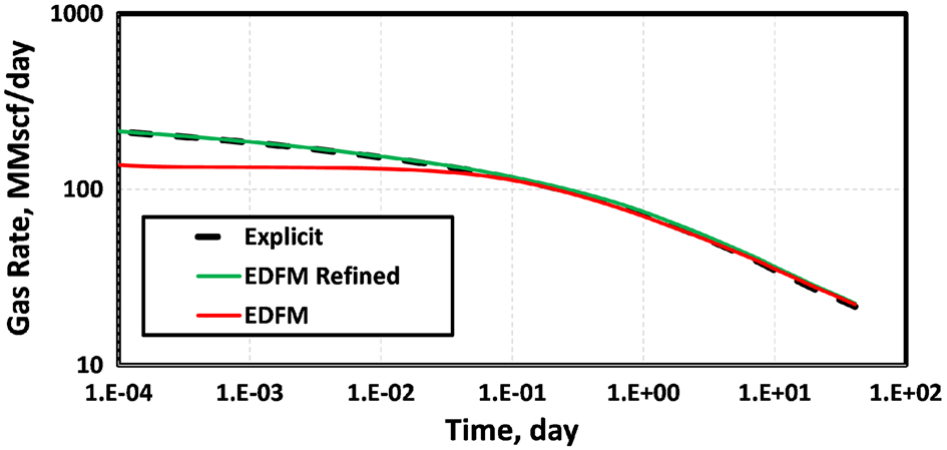
The gas production rates show that the EDFM case with a geometrically spaced mesh matches the reference solution from the explicit or fully dimensional model. When EDFM is used with structured Cartesian mesh, it underestimates the early-time production rate by up to 36%.

To obtain the results shown in Fig. 4, we used a three-component mixture of methane (C_1_), ethane (C_2_) and propane (C_4_) at mole-fractions of 0.991, 0.0088 and 0.0002, respectively. Table 1 summarizes the parameters of the reservoir simulated. Even though EDFM is used in the geometrically spaced (referred to as “EDFM Refined”) and Cartesian grids, the results are different. This is because the refined EDFM case can account for transient flow using geometrically spaced grids that are very fine near the fracture surface. In contrast, using EDFM in structured Cartesian grids without refinement cannot capture the expected transient flow at early times. This explains why the “EDFM Refined” case in Fig. 4 matches the reference solution given by the explicit model, whereas the EDFM case does not match it at early times.

**Table 1:** Reservoir model parameters.

Parameters	Field Unit	SI Unit
Initial reservoir pressure, P$_i$	5,700 psia	39.3 MPa
Reservoir temperature, T	352.6 K	175 °F
Wellbore flowing pressure, P$_{wf}$	1,000 psia	6.9 MPa
Fracture half-length, $x_f$	350 ft	106.7 m
Fracture width, $w_f$	0.01 ft	0.003 m
Fracture permeability, $k_f$	50 D	4.9 e−11 m^2^
Fracture porosity, $\phi _{f}$	0.5	0.5
Matrix permeability, $k_m$	100 μD	9.87 e−17 m^2^
Matrix porosity, $\phi _m$	0.16	0.16
Well radius, $r_w$	0.25 ft	0.076 m

Although EDFM is computationally efficient because it allows us to mesh the fractures independently of the matrix, the errors at early times limit its use without mesh refinement. Additionally, using mesh refinement with EDFM curtails its computational appeal because it will require refining all the matrix meshes near all the fractures in the simulation domain. To address this EDFM limitation, we propose an analytical EDFM modification, which is applicable in structured Cartesian grids without mesh refinement.

## EDFM modification for transient flow

This section shows how we modify the matrix/fracture non-neighboring connection in EDFM to account for transient flow between a tight host matrix and a fracture. The goal here is to find an analytical expression for a transient factor that applies to both early and late times, as Zimmerman et al.^13^ did for the transient matrix/fracture coupling term in the dual-continuum model. Comparing Eqs. (2) and (4) reveals that these two equations are essentially the same but with different definitions of the terms in the equation. In Eq. (2), the matrix/fracture NNC area ($A_m^{nnc}$) and distance ($d_m^{nnc}$) refer to the total surface area of the fracture surface within a host matrix, and the volume-weighted distance between the host matrix and the fracture, respectively. However, in Eq. (4), the area ($A_i$) and distance ($d_i$) refer to the matrix cell surface area and the distance between the centroid of the matrix and fracture cells, respectively. It is worth noting that $A_m^{nnc}$ and $d_m^{nnc}$ are summed over all the non-neighboring matrix/fracture connections, whereas $A_i$ and $d_i$ are summed over the number of faces that bound a matrix cell. Another difference in the terms in these two equations is that $k_m^{nnc}$ refers to the harmonic average of the matrix and fracture permeability in Equation (2), whereas $k_m$ in Eq. (4) refers to the matrix permeability.

Given the similarity between these two pseudo-steady state matrix/fracture non-neighboring or coupling flux terms, we modified Eq. (2) in the same manner that Eq. (4) was modified in Zimmerman et al.^13^. This modification allows us to account for the transient flow expected between tight matrix and fracture cells. The derivation of the transient shape factor in Zimmerman et al.^13^ shows that the pseudo-steady state matrix/fracture non-neighboring connection in Eq. (2) needs to be multiplied by the transient factor given in Eq. (8). So, the modified transient matrix/fracture NNC flux term is given as:

9 $$\begin{aligned} q_{i,T}^{nnc} = T_f \frac{1}{V}\sum _{m=1}^{N_{nnc}} A_m^{nnc}\sum _{\alpha =1}^{n_p}\frac{k_m^{nnc} k_{r\alpha }}{\mu ^\alpha }\rho ^\alpha X^\alpha _i\left( \frac{\Phi - \Phi _m^{nnc}}{d_m^{nnc}}\right) , \end{aligned}$$

where

10 $$\begin{aligned} T_f = \frac{2 \Phi _i - \left( \Phi _m + \Phi _f\right) }{2\left( \Phi _i-\Phi _m\right) }. \end{aligned}$$

Here, $\Phi _i$, $\Phi _m$, and $\Phi _f$ refer to the matrix or fracture flow potential at initial conditions, matrix flow potential at current conditions, and fracture flow potential at current conditions, respectively. Considering that $\Phi _m$ will be equal to $\Phi _i$ at the first time step and first Newton-Raphson iteration, we simply reduce $\Phi _m$ by 1x10$^-6 \%$ when $\Phi _m = \Phi _i$ to account for the edge effect.

Comparing Eq. (9) to Eq. (7) clearly shows that the standard EDFM model implicitly uses a pseudo-steady state (PSS) matrix/fracture flux term. In contrast, the proposed modification multiplies the PSS flux term by the transient factor ($T_f$) to obtain the transient matrix/fracture flux term. To use the proposed “transient” embedded discrete fracture model (tEDFM) instead of the standard EDFM, we simply substitute Eq. (9) (instead of Eq. (2)) into Eq. (1). To verify the accuracy, applicability, and computational efficiency of tEDFM, we implemented the proposed modification in our open-source “shale” module (https://github.com/UnconvRS/shale), which is one of the modules in MRST. We used MATLAB^22^ and MRST 2021b for all the simulations perfomed in this work. The next two sections discuss the validation and applications of tEDFM, respectively.

## Validation of tEDFM

This section focuses on validating the proposed EDFM modification by comparing it to reference solutions that can account for the expected transient flow between a tight rock matrix and fractures. The results presented in Fig. 3 are modified to include a case where the proposed EDFM modification is applied to the case with the structured Cartesian mesh.

**Figure 5:**
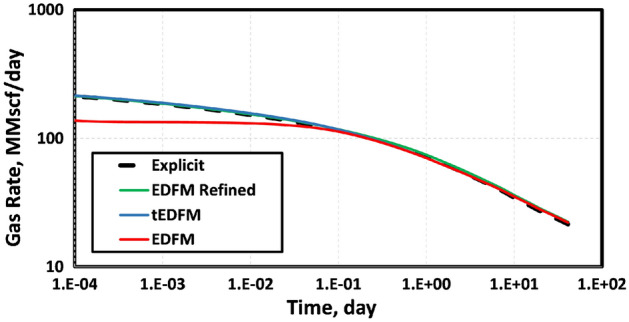
The gas production rates show that EDFM matches the reference solution when the modification presented is applied to the structured Cartesian mesh.

The solid blue line in Fig. 5 shows the simulation results obtained when the proposed modification is applied to the case with the structured Cartesian grid blocks (red line). The only difference between the model used to generate the blue and red lines in Fig. 5 is the inclusion of the transient flow modification. So, we conclude that the inability of the standard EDFM model to capture the early-time flow behavior is because of its implicit use of a pseudo-steady state matrix/fracture NNC instead of a transient one. In the last section of this work, we will confirm this conclusion using a more detailed analysis of a representative naturally fractured shale-oil reservoir.

**Figure 6:**
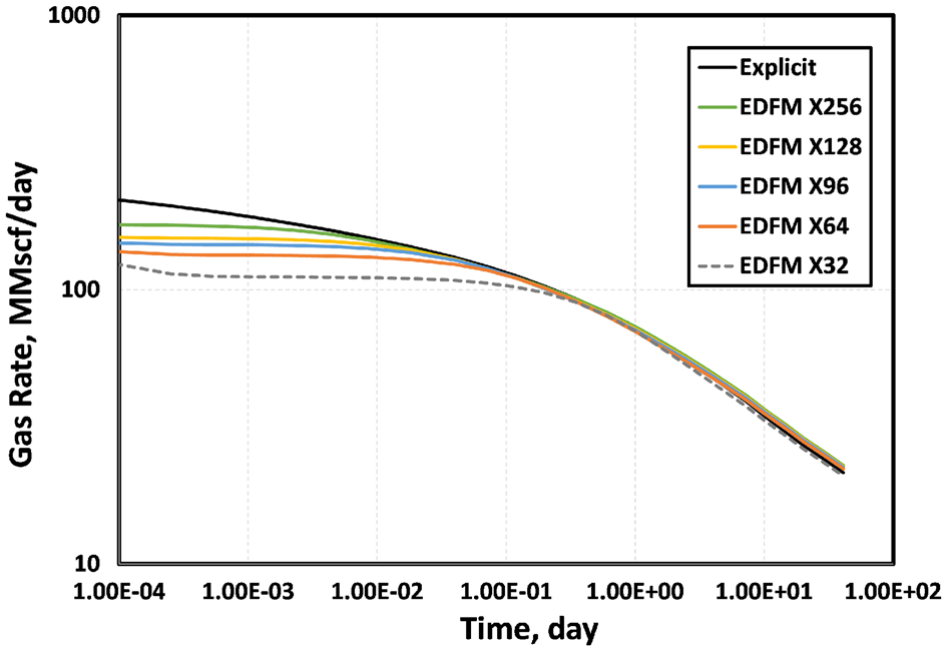
The results in this plot show that the standard EDFM is unable to match the early-time gas rates unless the cells in the structured Cartesian mesh are very small. The error increases as the meshes get more coarse.

**Figure 7:**
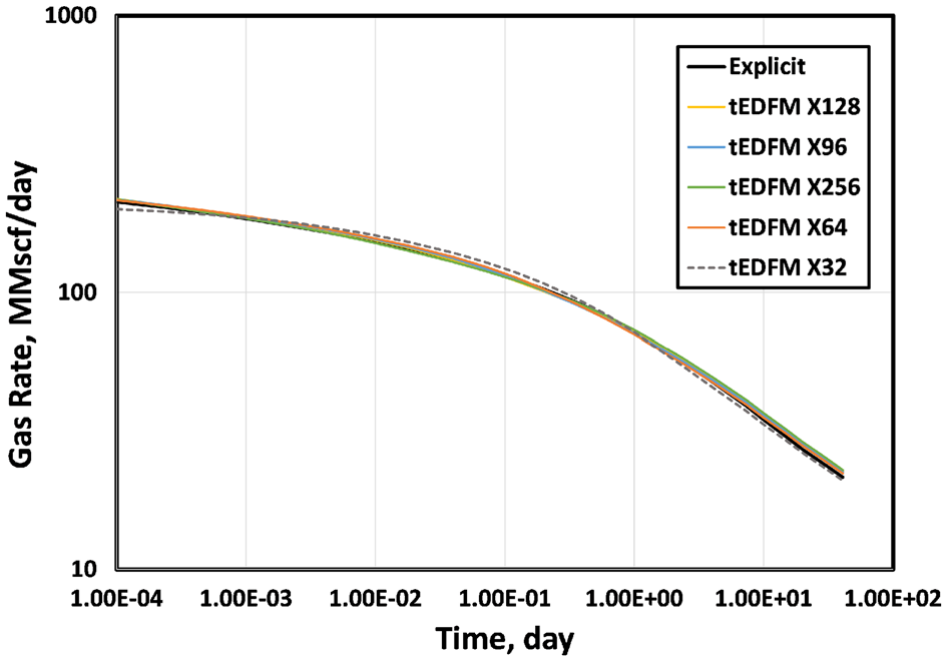
The results show that tEDFM is less sensitive to the mesh size.

Figures 6 and 7 show the sensitivity of the simulated gas rates to the sizes of the structured Cartesian cells. In both figures, we present the reference solution from the explicit model based on geometrically spaced grids that can account for the early-time transient flow. We use structured Cartesian meshes that are increasingly finer in both figures. The most refined mesh contains 256 matrix cells in the X-direction, while the coarsest has only 32 matrix cells in this direction. Fig. 6 shows that the standard EDFM cannot match the gas rates at early times, even with the most refined Cartesian mesh used. By including the modification presented, even the coarsest Cartesian mesh with only 32 matrix cells in the X-direction can match the reference solution from the explicit model. As expected, the error in matching the early-time rates increases as the mesh gets coarser. In contrast, Fig. 7 shows that tEDFM is less sensitive to the mesh size, particularly at early times.

**Figure 8:**
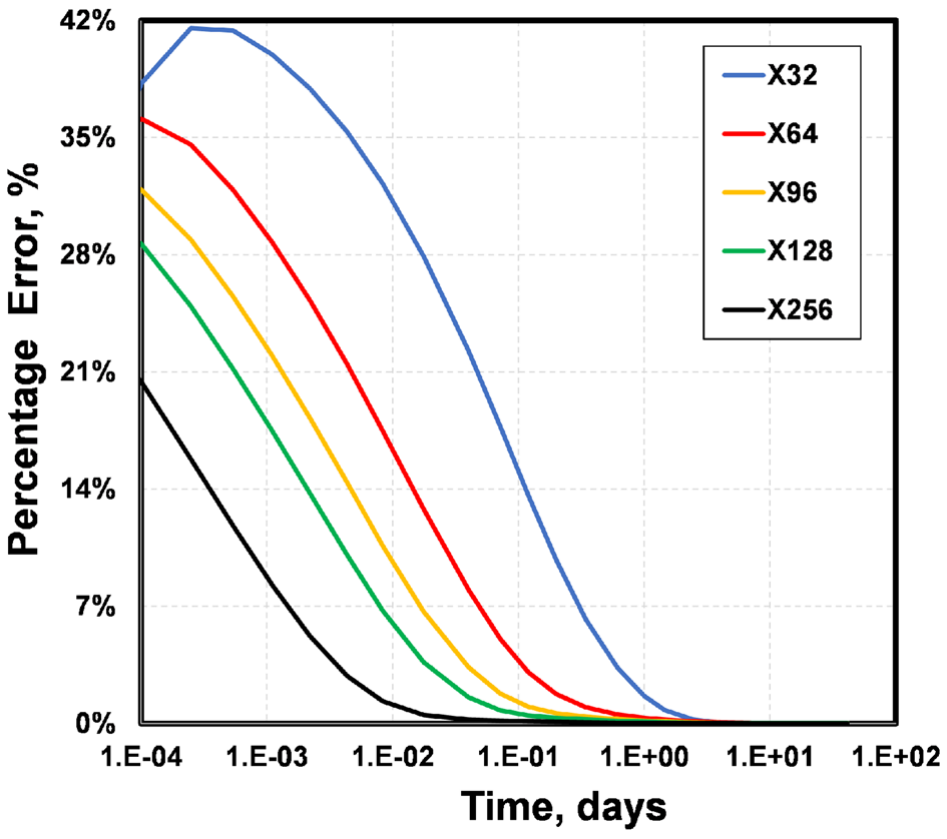
The results show that the percentage error in the standard EDFM can be up to 42%, depending on how coarse the structured Cartesian mesh is.

To quantify the error in the standard EDFM relative to tEDFM, we compute the percentage error in Fig. 8 as the ratio of the difference between both models’ rates to that of tEDFM. The results show that the error decreases as the mesh gets finer. This is expected because truncation error typically reduces as the mesh becomes more refined. However, even at the finest mesh size, the standard EDFM still has up to 20% error. This error increases up to 40% when the coarsest Cartesian mesh is used. It is worth noting that the truncation error in the coarsest grid with only 32 matrix cells in the x-direction is higher than in the other grids, resulting in a slightly different trend in Fig. 8. Therefore, a mesh with at least 64 matrix cells in the x-direction is required to obtain accurate solutions. The inaccuracy of this case can also be observed in its deviation from the reference solution shown in the mesh sensitivity presented in Fig. 7.

**Figure 9:**
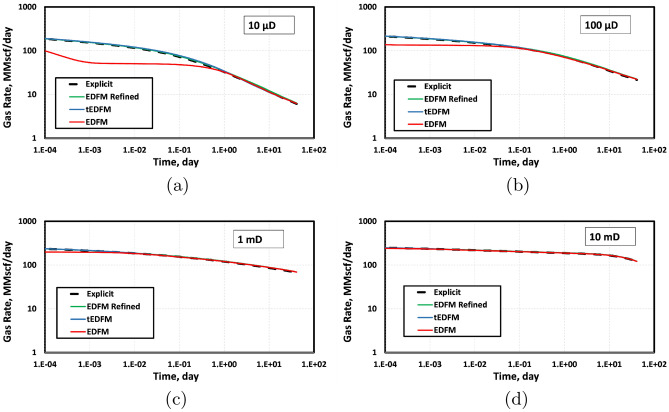
The gas production rates show that the standard EDFM error is more significant at lower matrix permeability values, while tEDFM matches the reference solution in all cases.

To show that the proposed transient EDFM modification is more important in tight rocks than in conventional reservoirs, we simulated all the cases presented in Fig. 5 at different permeability values. The results are presented in Fig. 9. The base case presented in Fig. 5 was simulated with a matrix permeability of 100 μD, so it corresponds to the top right plot in Fig. 9. This figure shows that the difference between the simulations based on EDFM and tEDFM is negligible when the permeability is greater than 1 μD. However, the error introduced by the pseudo-steady state assumption in the matrix/fracture NNC flux term becomes more significant at lower matrix permeability values. This indicates the importance of using tEDFM instead of EDFM in tight rocks.

## Application of tEDFM in the bakken shale

**Figure 10:**
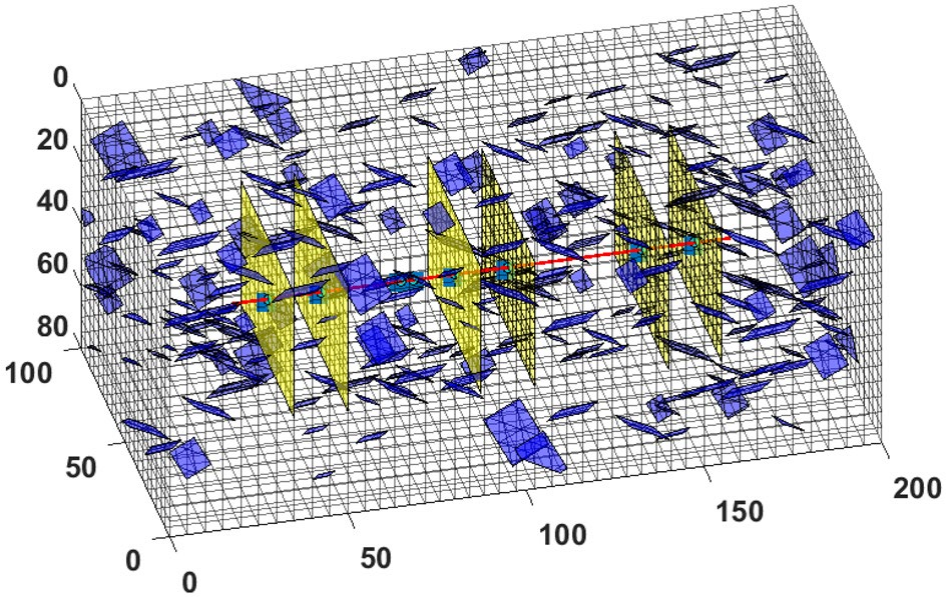
Image shows the simulation domain for modeling CGEOR in the Bakken shale play. The natural fractures are shown as blue planes, while the hydraulic fractures are the yellow vertical planes.

This section discusses the simulation of primary recovery followed by enhanced oil recovery in a volatile Bakken shale-oil reservoir. The goal is to evaluate the importance of the proposed modification and its computational efficiency. We use the fluid composition data presented Appendix-B , which are representative of the Bakken shale play. Table 2 summarizes the reservoir and fracture parameters used. The fractured reservoir simulated contains 256 natural fractures with dips ranging from 60° to 90° (with a mean of 80°). The dip direction varies between N 50° W and S 40° E, as interpreted from formation micro-imager (FMI) logs^23^. Figure 10 presents the simulation domain for the representative Bakken shale reservoir studied. The red horizontal line in the middle of the domain represents the horizontal well, while the hydraulic and natural fractures are shown as yellow and blue planes, respectively.

**Table 2:** Input parameters for CGEOR in Bakken Shale.

Parameters	Field Unit	SI Unit
Initial reservoir pressure, P$_i$	6,700 psia	46.2 MPa
Wellbore flowing pressure, P$_{wf}$	3,000 psia	20.7 MPa
Bubble-point pressure, P$_{b}$	1,640 psia	11.3 MPa
Reservoir temperature, T	352.6 K	175 °F
Fracture half-length, $x_f$	98.4252 ft	30 m
Fracture width, $w_f$	0.00984 ft	0.003 m
Cluster spacing	49.21 ft	15 m
Fracture spacing	123.03 ft	37.5 m
Fracture permeability, $k_f$	1 D	9.87 e−13 m^2^
Fracture porosity, $\phi _f$	0.5	0.5
Matrix permeability, $k_m$	100 nD	9.87 e−20 m^2^
Matrix porosity, $\phi _m$	0.07	0.07
Well radius, $r_w$	0.32 ft	0.1 m
Injection rate	60 Mscf/day	0.02 m^3^/s
Injection period	60 days	5.184e+6 s
Soaking period	14 days	1.21e+6 s
Production period	180 days	15.55e+6 s
Cycle duration	254 days	21.95e+6 s

**Figure 11:**
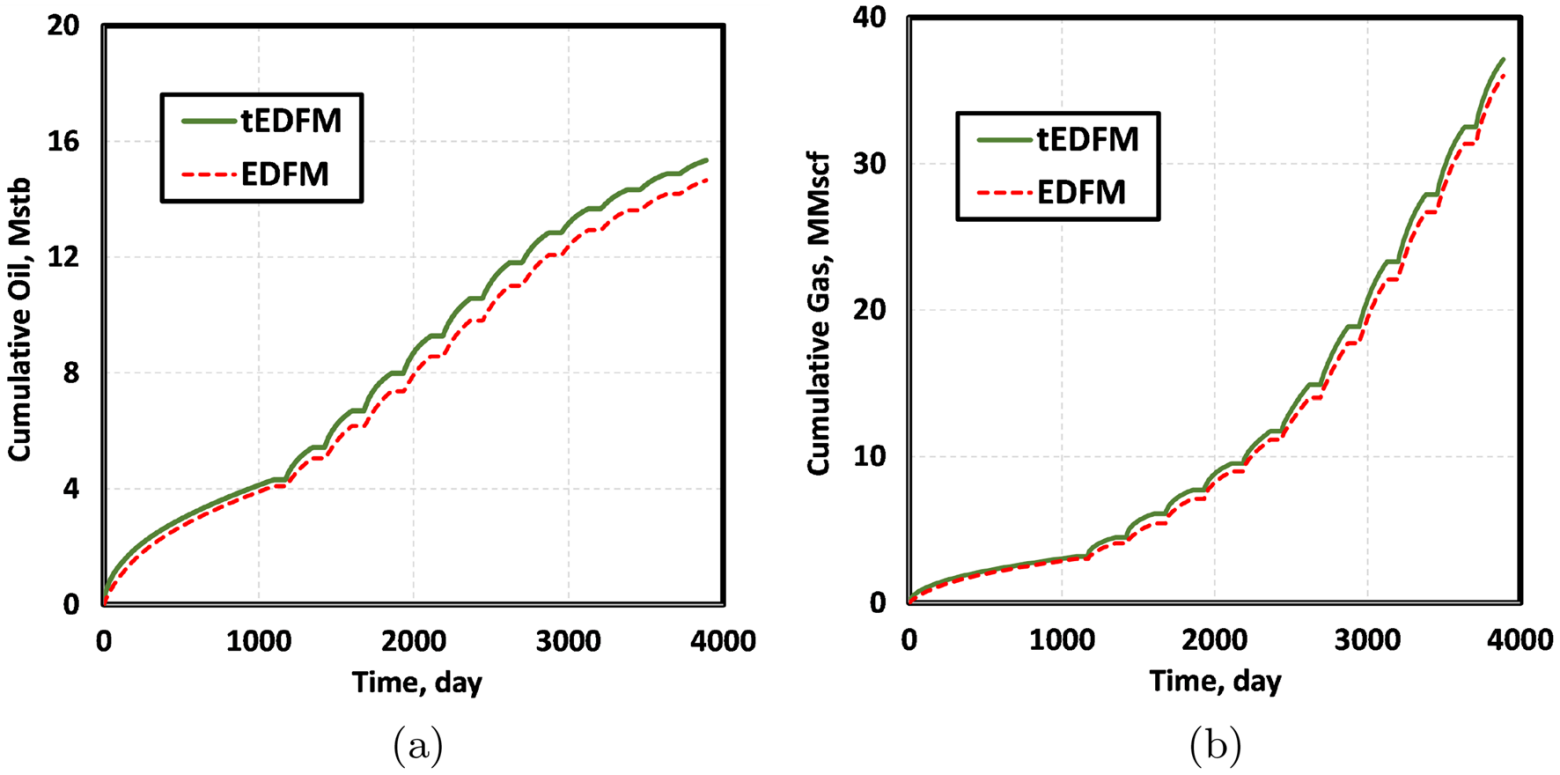
The cumulative oil and gas production shows the effect of the modification. Without the modification, the oil and gas production volumes are underestimated by 4% and 3% respectively in HnP simulation.

We simulate the cyclic injection of methane for eight more years after three years of primary production. The injection, soaking, and production duration shown in Table 2 are consistent with the optimum field operating conditions used in Kuuskraa et al.^24^. Figure 11 presents the cumulative oil and gas production obtained using EDFM and tEDFM models. The results show that EDFM underestimates the cumulative oil and gas production by 4% and 3%, respectively. This is because it cannot capture the transient flow, which is very significant at the beginning of the primary production and the production periods after the injection and soaking periods.

**Figure 12:**
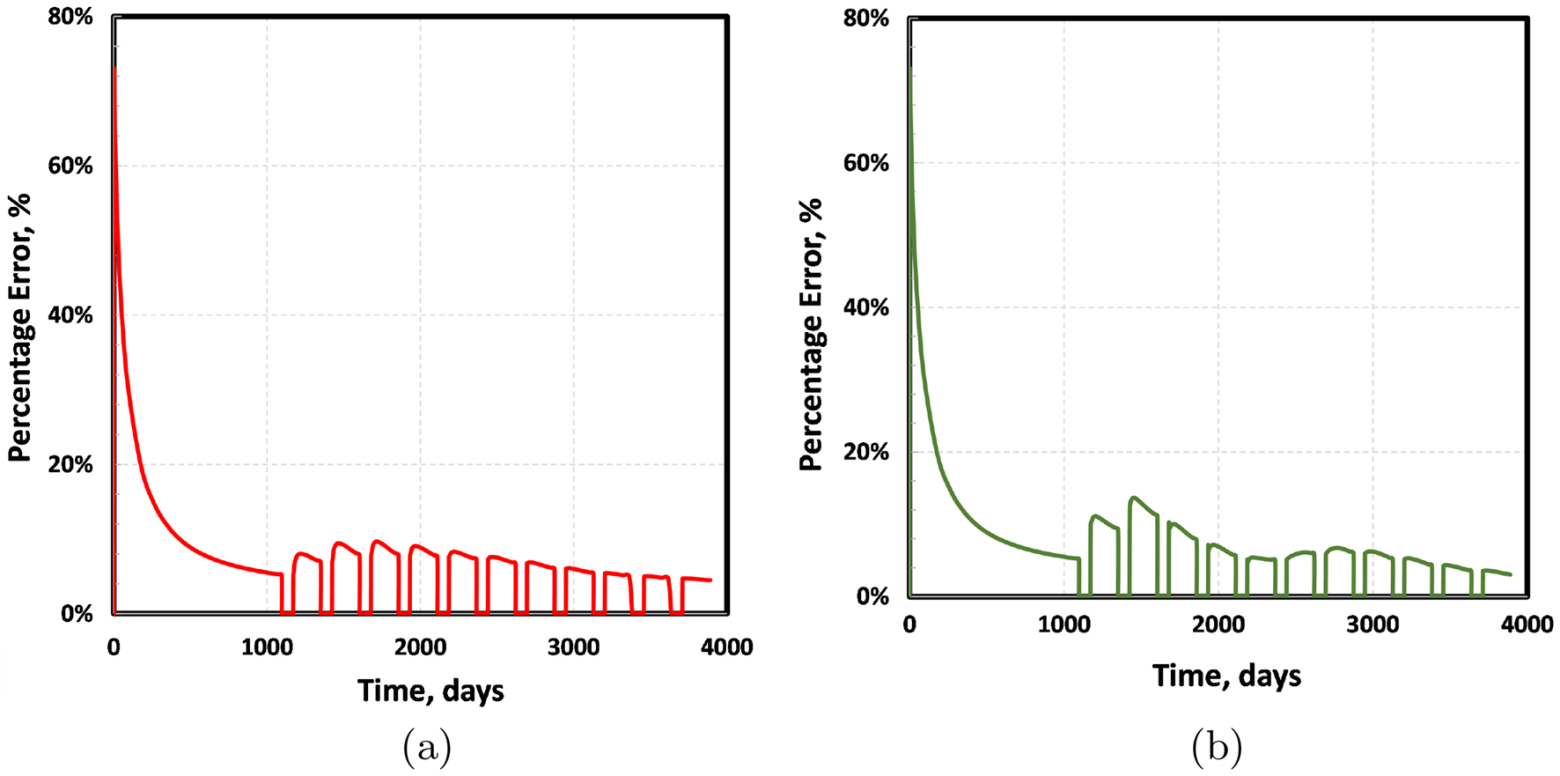
Left plot shows the percentage error when the EDFM cumulative oil production is compared to that of tEDFM. The right plot shows a similar percentage error comparison in cumulative gas production.

The left and right images in Fig. 12 correspond to the percentage errors of EDFM (relative to tEDFM) based on the cumulative oil and gas production, respectively. Although the standard EDFM error (relative to tEDFM) at the end of the simulation is only 4% and 3% for the oil and gas cumulative production, the results show that the error is more significant during the life of the well. The figure shows that the percentage error for the cumulative oil production is up to 73% at very early times but declines exponentially to as low as 5% after three years of primary oil production. Over the next three years of cyclic gas enhanced oil recovery, this percentage error increases to 10% after 5 years; then, it gradually declines to 4% at the end of the simulation (11 years). The right plot in Fig. 12 also shows a similar trend, where the percentage error in the cumulative gas production declines exponentially from 73% to 5% after three years of primary oil production. Over the next three years of cyclic gas enhanced oil recovery, this percentage error increases to 14% after 5 years before gradually declining to 3% at the end of the simulation.

**Figure 13:**
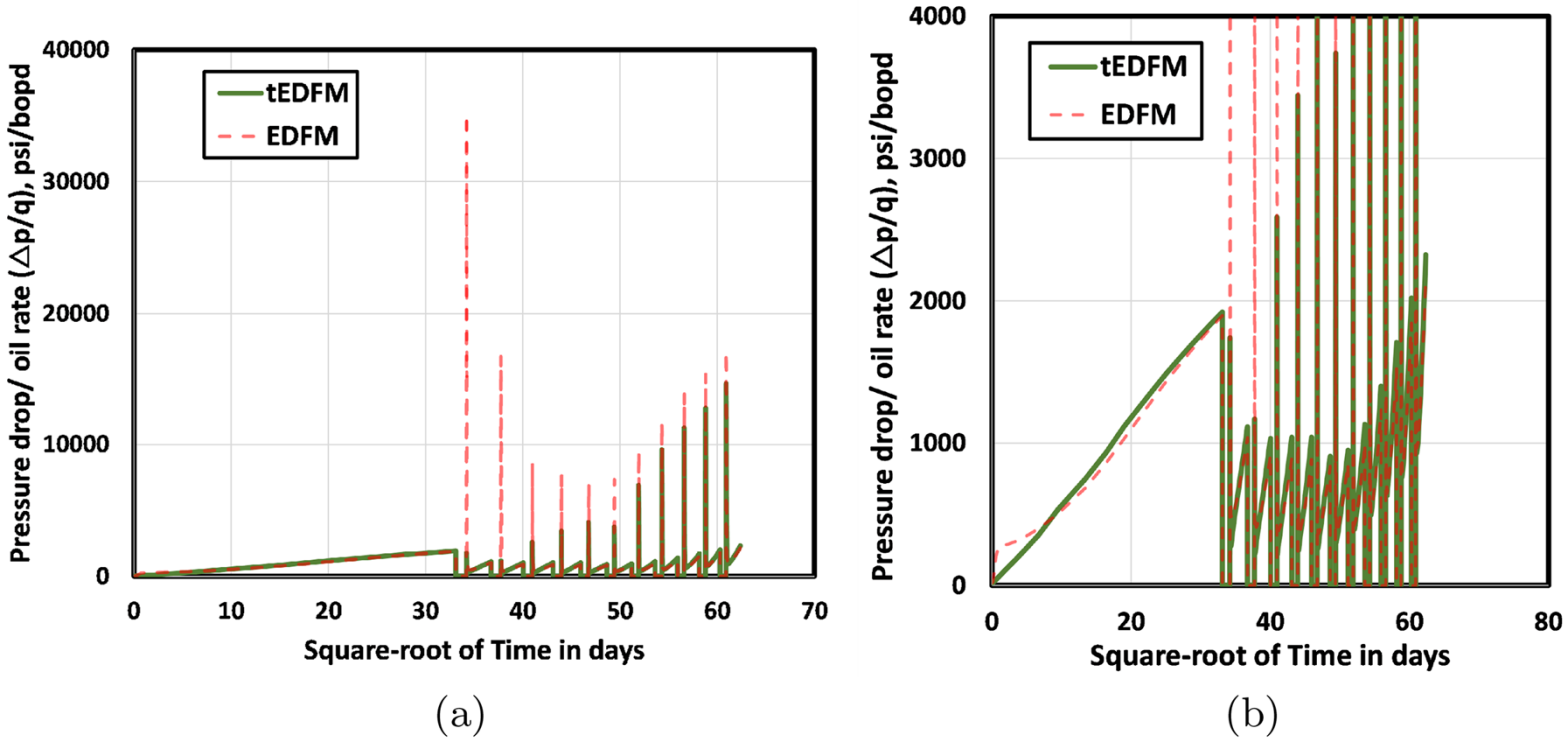
Both plots in this figure compare the EDFM and tEDFM model results using square-root-time RTA plots. The left plot shows the full range of the results, while the right plot reduces the upper y-axis limit to focus on the expected early-time linear trend that is well captured in tEDFM but not in EDFM.

Although the previous results already show the limitations of the standard EDFM, here we seek to evaluate both EDFM and tEDFM in terms of the expected flow signatures on standard rate-transient analysis (RTA) plots. Figure 13 presents the square-root time (rate-normalized pressure drop versus square-root time) plot for both EDFM and tEDFM. The left plot shows the full scale of the result, whereas the right plot fixes the upper limit of the y-axis at 4,000 psi/bopd. This lets us see the expected linear trend in the tEDFM results. In contrast, the EDFM model results show an erroneous nonlinear trend that becomes linear only after a value of 9.5 on the x-axis of Fig. 13b. Squaring this value indicates that the EDFM model transitions from the early-time erroneous results to the expected linear trend after 90 days of production. This result confirms the conclusion that EDFM is inaccurate at early times because of its implicit pseudo-state flow assumption in the matrix-fracture transfer term. The fact that the tEDFM model shows a clear linear trend even at early times indicates that the simple analytical modification presented fixes this limitation of the standard EDFM model.

**Figure 14:**
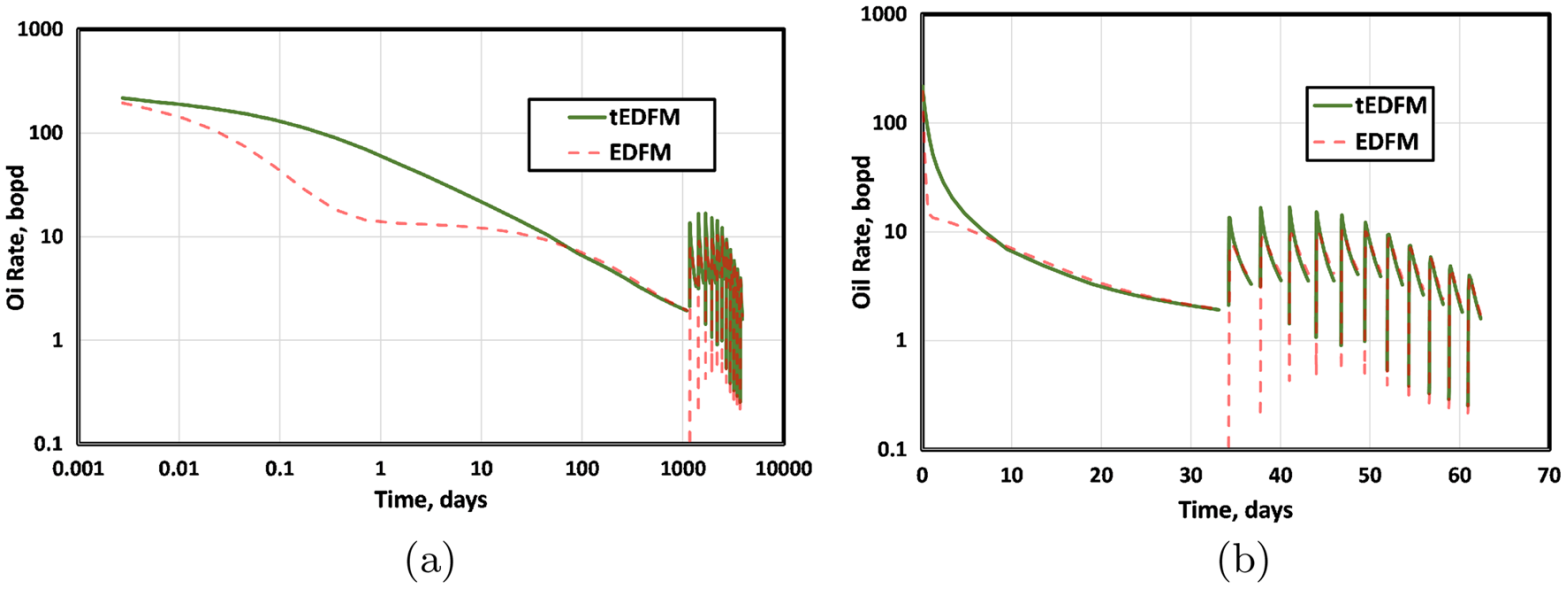
This figure presents a comparison of tEDFM and EDFM oil rates using (**a**) log-log oil rate versus time and (**b**) semi-log oil rate versus time plots. The tEDFM results show the expected profiles in tight rocks, whereas EDFM results appear erroneous at early times.

**Figure 15:**
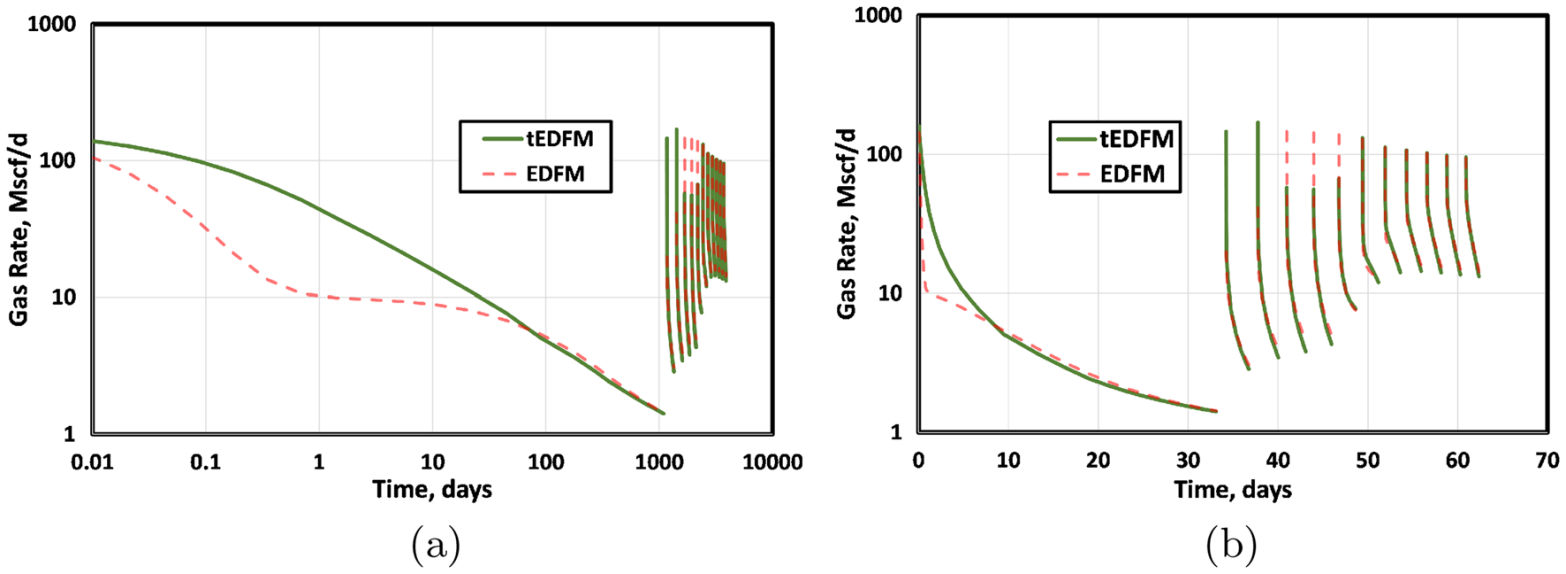
This figure presents a comparison of tEDFM and EDFM gas rates using (**a**) log-log gas rate versus time and (**b**) semi-log gas rate versus time plots. The tEDFM results show the expected profiles in tight rocks, whereas EDFM results appear erroneous at early times.

To further show the difference between the EDFM and tEDFM models at early times, Fig. 14 presents the log-log and semi-log plots of the oil rate, while Fig. 15 presents the corresponding gas-rate plots. Considering that log-log plots tend to exaggerate the early-time differences, the left plot in Figs. 14 and 15 more clearly show that the tEDFM model is consistent with typical oil and gas rate trends expected from tight rocks. In contrast, the corresponding EDFM oil and gas-rate log-log plots show an observable deviation from the expected trends observed from the high-resolution reference solutions presented in the previous section (e.g., Fig. 9a for tight rocks). The right plots in Figs. 14 and 15 show unrealistically sharp drops in the oil and gas rates obtained from EDFM, whereas the corresponding tEDFM results show more realistic oil and gas rate declines. These results show that the proposed modification to the standard EDFM gives more realistic forecasts of oil and gas production, especially at early times.

**Figure 16:**
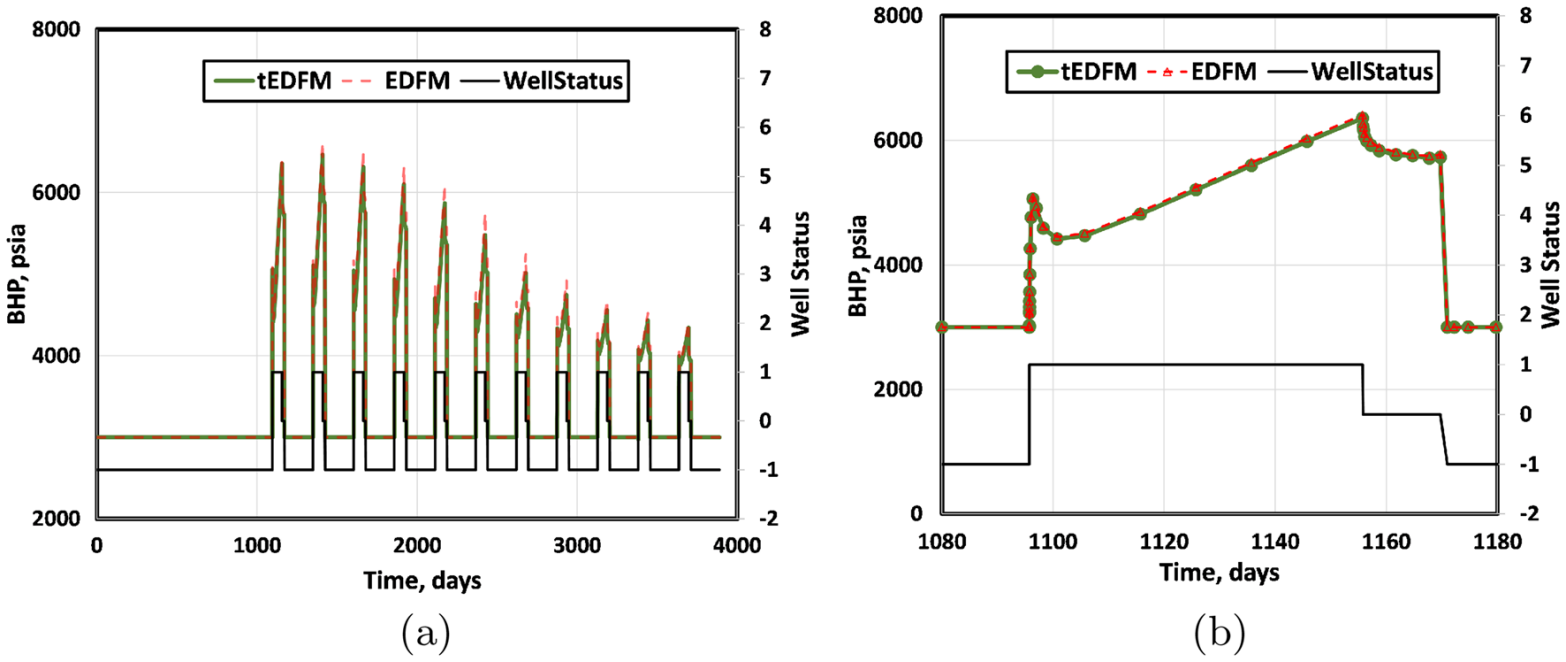
The left plot shows the full range of the BHP over the simulated time frame, while the right plot only focuses on the first injection, soaking, and production cycle. The results show a negligible difference between the tEDFM and EDFM BHPs.

Figure 16 presents the bottomhole pressures (BHP) corresponding to the EDFM and tEDFM models on the primary y-axis. The secondary y-axis shows the well status, which is the same for both EDFM and tEDFM. It is set to minus one (-1) during production, zero (0) when the well is shut in during the soaking period, and one (1) during gas injection. The left plot shows the full range of the model results, while the right plot zooms in on the first injection, soaking, and production cycle by constraining the x-axis between 1,080 and 1,180 days. The result shows that the BHP is constant at a value of 3,000 psi during the pressure-constrained production periods. The trend during the injection period is more complicated because the well transitions from a pressure-constrained producer to a rate-constrained injector. So, the BHP increases because of the higher pressures in the neighboring grid blocks during production and gas injection at a fixed rate. The result is a sharp increase in BHP followed by a short period of pressure drop, then a steady BHP increase. The BHP drops as expected when the well is shut in during the soaking period. This cycle is repeated as shown in Fig. 16a. Figure 16 shows little or no difference between EDFM and tEDFM in terms of the simulated flowing bottomhole pressures.

**Figure 17:**
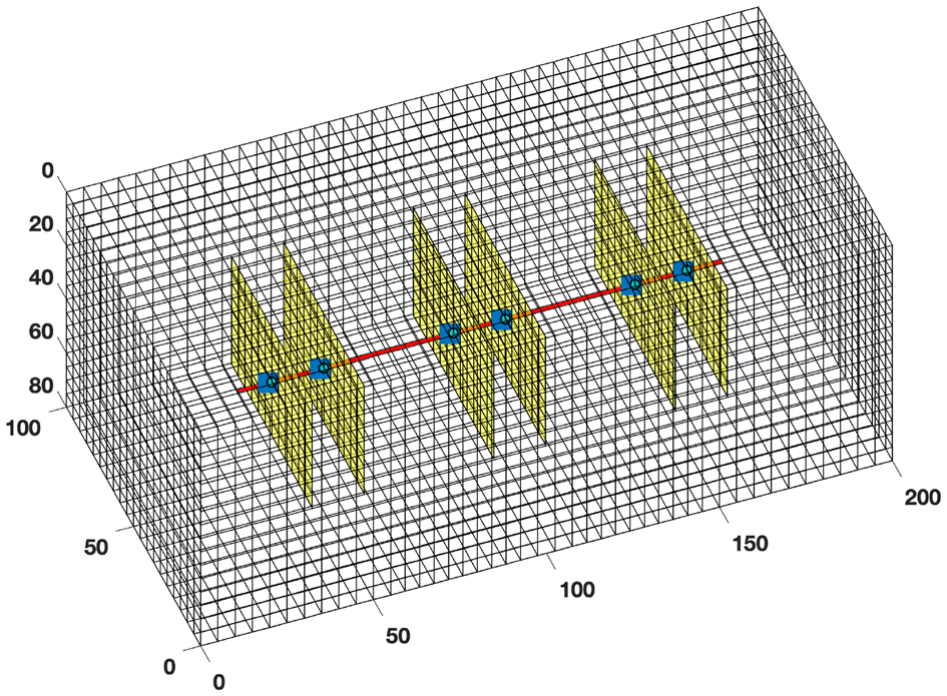
Cartesian grid for the Bakken shale play with hydraulic fractures only. The natural fractures are removed to allow us obtain reference solutions.

**Figure 18:**
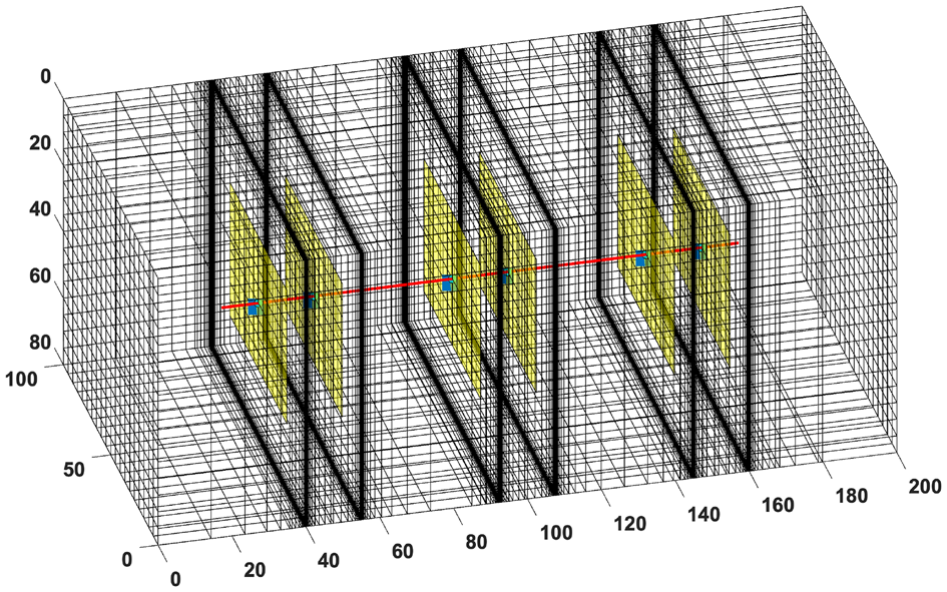
Image shows the logarithmmically spaced grid that is capable of capturing the transient flow near the fracture surfaces.

Considering the extreme computational challenge of obtaining high-resolution reference solutions for the naturally fractured system shown in Fig. 10, we simulated only the first three years of primary production, took out the natural fractures, and modeled the same system using Cartesian and logarithmically spaced grids. The idea is to obtain the reference solution for the system with only the hydraulic fractures and compare this to the results from using tEDFM and EDFM with Cartesian grids. Figures 17 and 18 provide the mesh for the Cartesian and logarithmically spaced grids, respectively. In Fig. 18, the mesh size for the finest matrix cells near the fracture surface is roughly the same size as the fracture width.

**Figure 19:**
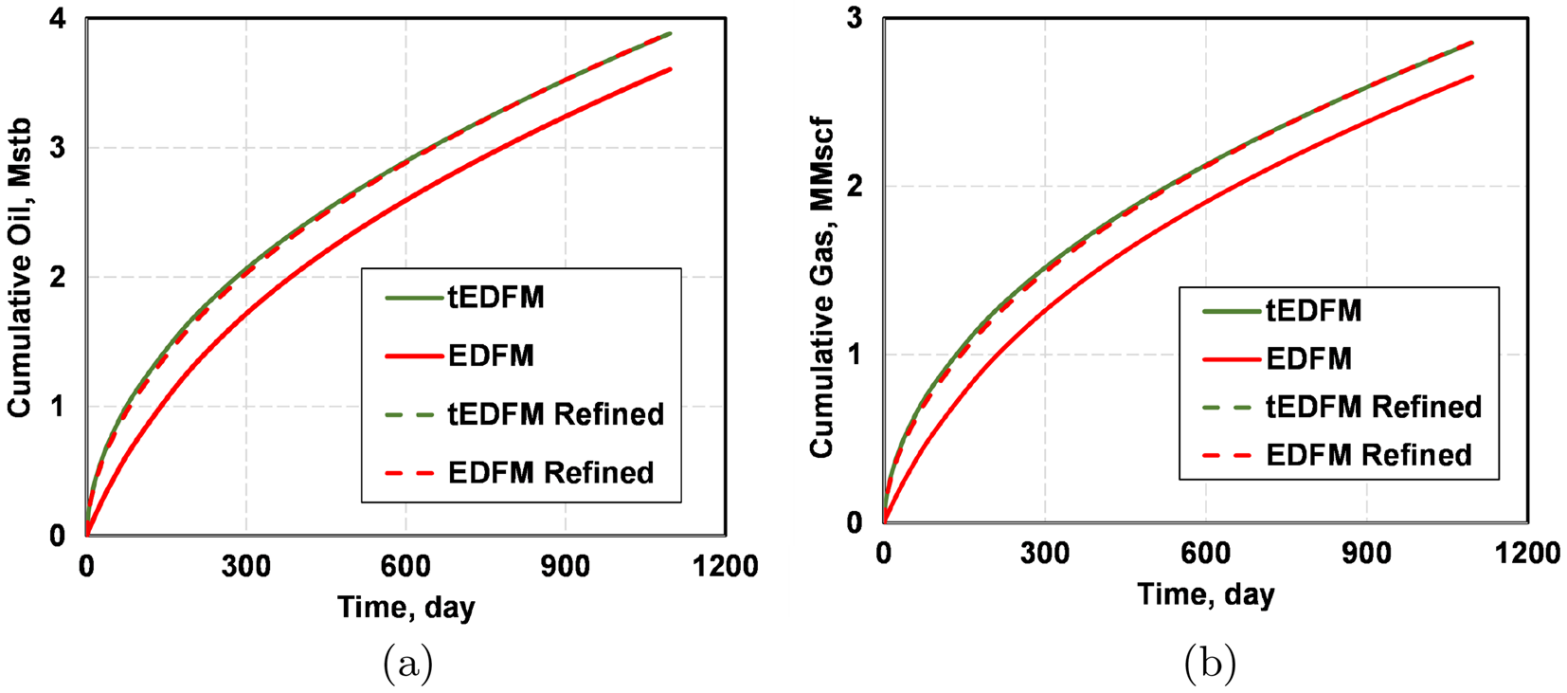
The plots in this figure show that EDFM underestimates the cumulative oil and gas production by 7.2%, whereas tEDFM only underestimates both the cumulative oil and gas production by 0.1%.

Figure 19 presents the plots of the cumulative oil and gas production when tEDFM and EDFM are used with the Cartesian and logarithmically spaced grids. These results show that both tEDFM and EDFM yield accurate results when using the logarithmically spaced grid. This is because the fine mesh near the fracture surface captures the expected transient flow in both models. However, when a Cartesian mesh with a constant mesh size is used in the domain, EDFM can no longer capture the transient flow, but tEDFM still does. This again points to the computational advantage and accuracy of tEDFM because it can capture the expected transient flow without requiring mesh refinement.

**Figure 20:**
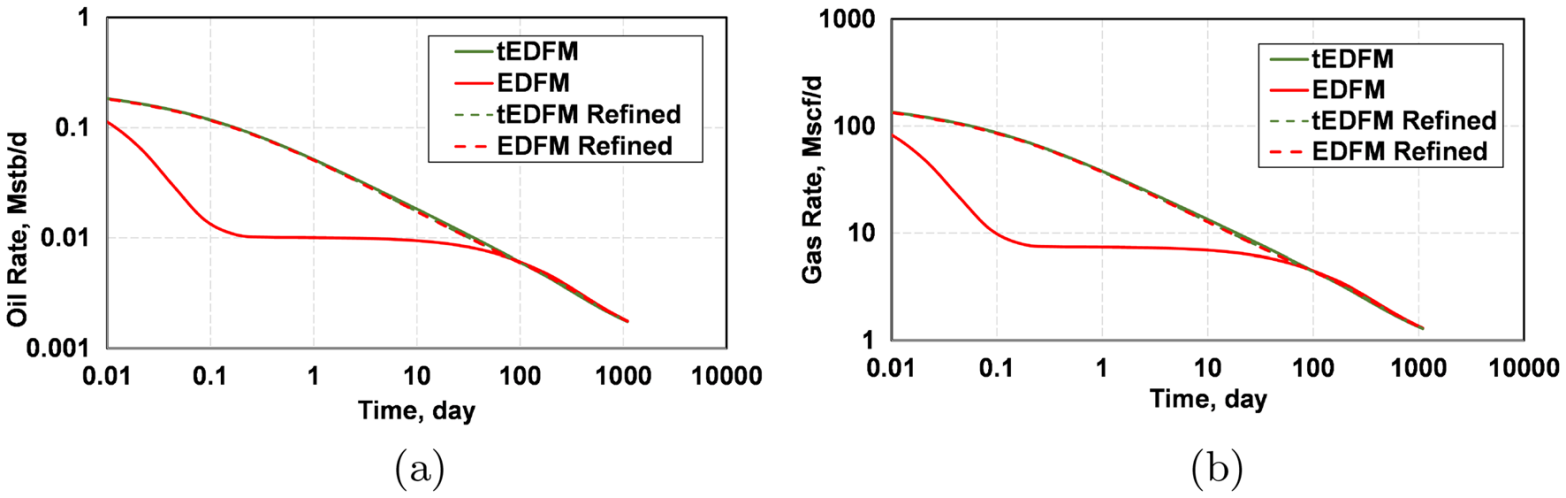
The plots of the oil and gas rates show that tEDFM matches the reference solutions from the refined log-spaced mesh, whereas EDFM does not.

Finally, Fig. 20 presents the plots of the oil and gas rates when EDFM and EDFM are used with the Cartesian and logarithmically spaced grids. Like the cumulative oil and gas production plots, these results show that tEDFM and EDFM yield accurate results when the logarithmically spaced mesh is used. However, EDFM yields erroneous results when the Cartesian mesh is used. The fact that the tEDFM result for the Cartesian mesh still matches the reference solution obtained from the logarithmically spaced grid confirms the applicability of the proposed model even with coarse Cartesian grids.

## Conclusions

This work presents a fast and accurate EDFM modification that addresses the limitation of EDFM in tight rocks with transient flow. Although EDFM can be used in structured Cartesian grids, we show that it yields inaccurate results in ultra-low permeability rocks at early times. By comparing EDFM to high-resolution reference solutions, we show that EDFM yields up to 73% error at early times when the cells in the domain are not small enough to capture the expected transient flow from the tight matrix into the fracture. This is because the standard EDFM implicitly assumes pseudo-steady flow between the matrix and fractures. To obtain a transient matrix/fracture NNC flux term that addresses the limitation of the standard EDFM, we propose the use of a dual-continuum type transient factor, as in Zimmerman et al.^13^. The proposed analytical modification yields a transient embedded discrete fracture model (tEDFM) that matches the high-resolution reference solutions presented at all (early and late) times.

The simulation results for the single vertical fracture cases show that we can match high-resolution, fully dimensional reference solutions using the proposed modification with coarse Cartesian grids. We also simulated cyclic gas enhanced oil recovery in a representative Bakken shale reservoir with 256 natural fractures. The results show that the proposed modification is robust enough for field studies and is only 1.5% slower than the standard EDFM for the naturally fractured Bakken shale reservoir simulated. It also shows that the standard EDFM underestimates the oil and gas production at the beginning of each production cycle. Finally, we presented several typical rate-transient analysis plots (such as square-root-time, log-log, and semi-log plots) that confirm the superior performance of the proposed transient EDFM model, especially at early times.

## Supplementary Information

Supplementary Information 1.

Supplementary Information 2.

## Data Availability

All data generated or analysed during this study are included in this published article and its supplementary information files.
