# Supplementary material for: Analytical modification of EDFM for transient flow in tight rocks
Source: Sci Rep. 2022 Dec 20;12:22018. doi: 10.1038/s41598-022-26536-w (PMC9768198; doi:10.1038/s41598-022-26536-w)
Supplement: Supplementary file 1 — Supplementary Information 1. [file 41598_2022_26536_MOESM1_ESM.pdf]

Appendix A Governing Equations for Compositional Reservoir Simulation

Without accounting for the presence of natural fractures, the governing equations for the mass conservation of each hydrocarbon component, i, in the liquid (l) and vapor (v) phases are given as:

$$\frac{\partial}{\partial t}\left(\phi[\rho^l S^l X_i^l + \rho^v S^v X_i^g]\right) + \nabla \cdot (\rho^l X_i^l \vec{v}^l + \rho^v X_i^g \vec{v}^v) - (\rho^l X_i^l q^l + \rho^v X_i^g q^v)/V = 0. \tag{A-1}$$

Similarly, the mass conservation equation for water (w) in the aqueous phase is given as:

$$\frac{\partial}{\partial t}(\phi \rho^w S^w) + \nabla \cdot (\rho^w \vec{v}^w) - \rho^w q^w/V = 0, \tag{A-2}$$

where ϕ, ρ^α, S^α, and q^α represent the matrix porosity, mass density, saturation, and volumetric withdrawal/injection rate of phase α, respectively. The symbols X_i^l and X_i^g represent the mass fractions of component i in the liquid and vapor phases, while $\vec{v_l}$ and $\vec{v_v}$ are the Darcy velocities for the liquid and vapor hydrocarbon phases, respectively. Note that the division of the source/sink term in Equations (A-1) and (A-2) by bulk volume, V is needed for dimensional consistency.

We obtain the phase velocities in Equations (A-1) and (A-2) from Darcy's equation as follows:

$$\vec{v}^\alpha = -\mathbf{K}\frac{k^\alpha(S)}{\mu^\alpha}(\nabla p^\alpha - \rho^\alpha g \nabla z), \tag{A-3}$$

where μ^α and $\mathbf{K}$ represent the phase viscosity and absolute matrix permeability, respectively. In the natural variables composition approach [1], which is used in this work, the primary variables are pressure, vapor and liquid composition of all but the last component, and water saturation (p, x_1^l,x_1^g ..., x_{n-1}^l, x_{n-1}^g, and S_w), respectively. The auxiliary thermodynamic equations and constraints needed for compositional simulation are summarized as follows:

$$f_i^g(p, T, y_1, \ldots, y_n) - f_i^l(p, T, x_1, \ldots, x_n) = 0, \quad \text{for } i \in 1, \ldots, n_c, \tag{A-4}$$

$$z_i - Lx_i - (1 - L)y_i = 0, \quad \text{for } i \in 1, \ldots, n_c, \tag{A-5}$$

$$\sum_{i=1}^{n_c} x_i = 1, \quad \text{for } i \in 1, \ldots, n_c, \tag{A-6}$$

$$\sum_{i=1}^{n_c} y_i = 1, \quad \text{for } i \in 1, \ldots, n_c, \tag{A-7}$$

$$S^w + S^l + S^v = 1.0. \tag{A-8}$$

In these equations, f_i^g and f_i^l are the fugacities of each component in the gas and liquid phases, respectively. Equation (A-4) ensures that the fugacity of each component in the vapor phase is equal to that of the same component in the liquid phase (which is required at chemical equilibrium), Equation (A-5) ensures that the sum of the number of moles of each component in the liquid and gas phases is equal to its corresponding overall composition, while Equations (A-6), (A-7), and (A-8) ensure that all mole fractions and saturations sum up to one.

We use the Peng-Robinson equation of state [2] to compute the fugacities and phase compressibility factors (Z^g and Z^l). Firoozabadi (2015) [3] provides more details on the equation of state, flash procedure, and the equations to compute the fugacities and compressibility factors. To solve the continuous equations in (A-1) and (A-2) numerically, we first perform a temporal discretization using the backward Euler scheme as follows:

$$\begin{aligned}\frac{1}{\Delta t}\left[\left(\phi\rho^l S^l X_i^l + \phi\rho^v S^v X_i^g\right)^{n+1} - \left(\phi\rho^l S^l X_i^l + \phi\rho^v S^v X_i^g\right)^{n}\right] + \\ \nabla\cdot(\rho^l X_i^l \vec{v}^l + \rho^v X_i^g \vec{v}^v) - (\rho^l X_i^l q^l + \rho^v X_i^g q^v)/V = R_i,\end{aligned} \tag{A-9}$$

$$\frac{1}{\Delta t}\left[(\phi\rho^w S^w)^{n+1} - (\phi\rho^w S^w)^{n}\right] + \nabla\cdot(\rho^w \vec{v}^w) - \rho^w q^w/V = R^w. \tag{A-10}$$

In the above equations, $n+1$ represents the current time step, while n represents the previous time step. Note that all other terms without these superscripts are evaluated at the current time step. We then discretize the flux terms in space using the Finite Volume Method (FVM) with a two-point flux approximation (TPFA). The TPFA method involves integrating Equations (A-9) and (A-10) over a control volume, after which the divergence theorem is applied. In this work, we use the discrete divergence (*div*) and gradient (*grad*) operators, which are discussed in the MATLAB reservoir simulation book [4] and implemented as functions in the MATLAB reservoir simulation toolbox (MRST). The resulting discretized form of Equations (A-9) and (A-10) can be written as:

$$\begin{aligned}\frac{V}{\Delta t}\left[\left(\phi\rho^l S^l X_i^l + \phi\rho^v S^v X_i^g\right)^{n+1} - \left(\phi\rho^l S^l X_i^l + \phi\rho^v S^v X_i^g\right)^{n}\right] \\ +\mathrm{div}(\rho^l X_i^l \vec{v}^l + \rho^v X_i^g \vec{v}^v)^{n+1} - (\rho^l X_i^l q^l + \rho^v X_i^g q^v)^{n+1} = R_i^{n+1},\end{aligned} \tag{A-11}$$

and

$$\frac{V}{\Delta t}\left[(\phi\rho^w S^w)^{n+1} - (\phi\rho^w S^w)^{n}\right] + \mathrm{div}(\rho^w \vec{v}^w)^{n+1} - (\rho^w q^w)^{n+1} = R_w^{n+1}, \tag{A-12}$$

where

$$\vec{v}^{\alpha} = -T_{ik}\lambda_{\alpha}^{n+1}\left[\text{grad}(p_{\alpha}^{n+1}) - \rho_{\alpha}^{n+1} g \, \text{grad}(z)\right], \tag{A-13}$$

$$T_{ik} = \left[T_{i,k}^{-1} + T_{k,i}^{-1}\right]^{-1}, \tag{A-14}$$

and

$$T_{i,k} = A_{i,k}\mathbf{K}_i \frac{\vec{c}_{i,k} \cdot \vec{n}_{i,k}}{\left\|\vec{c}_{i,k}\right\|^2}. \tag{A-15}$$

Here, V and $A_{i,k}$ refer to the cell volumes and face areas, respectively. The symbol, $\vec{n}_{i,k}$ is the unit normal in the direction from the centroid of cell, i towards the face between cells i and k, while $\vec{c}_{i,k}$ is the vector from the cell centroid to the face centroid. Additionally, T_{ik} is face transmissibility, whereas $T_{i,k}$ is the contribution of a cell to the face transmissibility. This transmissibility ($T_{i,k}$) is referred to as a half-transmissibility because a pair of cells contributes to the transmissibility of each face in the TPFA formulation. Note that the temporal and spatial discretizations of the continuous partial differential equations lead to a mass imbalance, which is represented by the residual (R) in Equations (A-9) through (A-12). The Newton-Raphson method involves applying the Taylor expansion to the residual at the current time step and current Newtonian iteration to obtain:

$$\frac{\partial R^{k+1}}{\partial X}\Delta X = -R^{k+1}(X), \tag{A-16}$$

where X denotes the primary variables. The matrix that contains the partial derivatives of the residuals with respect to each of these primary variables ($\frac{\partial R^{k+1}}{\partial X}$) is referred to as the Jacobian matrix. The setup of this matrix is facilitated using automatic differentiation in MRST, and more details on the solution of the system of equations for compositional flow are provided in Møyner et al (2017) [5]. Considering that most shale/tight oil reservoirs are naturally fractured to some extent, this work will involve simulating the proposed SDEOR method in such reservoirs with or without natural fractures. The next section explains how the discretized governing equations are modified to model natural fractures accurately and efficiently.

Appendix B Compositional Fluid Data

Tables B-1 and B-2 provide the compositional fluid data and binary interaction coefficients for a representative Bakken shale-oil reservoir.

Table B-1: Compositional data for Bakken light oil shale formation, culled from Yu et al (2015) [6].

Components	Mole Fraction	Critical Pressure (atm)	Critical Temperature (K)	Critical Volume (L/mol)	Molar Weight (g/gMol)	Acentric Factor	Parachor Coefficient
CO_2	0.0002	72.80	304.20	0.0940	44.01	0.225	78.0
N_2	0.0004	33.50	126.20	0.0895	28.01	0.040	41.0
C_1	0.25	45.40	190.6	0.0990	16.04	0.008	77
C_2-C_4	0.22	42.54	363.30	0.1970	42.82	0.143	145.2
C_5-C_7	0.20	33.76	511.56	0.3338	83.74	0.247	250
C_8-C_9	0.13	30.91	579.34	0.4062	105.91	0.286	0.099
C_{10+}	0.1994	21.58	788.74	0.9208	200.00	0.686	0.099

Table B-2: Binary Interaction Coefficients for Bakken light oil shale formation, culled from Yu et al (2015) [6].

Component	CO_2	N_2	C_1	C_2-C_4	C_5-C_7	C_8-C_9	C_{10+}
CO_2	0	0.02	0.1030	0.1327	0.1413	0.15	0.15
N_2	0.02	0	0.013	0.0784	0.1113	0.12	0.12
C_1	0.1030	0.013	0	0.0078	0.0242	0.0324	0.0779
C_2-C_4	0.1327	0.0784	0.0078	0	0.0046	0.0087	0.0384
C_5-C_7	0.1413	0.1113	0.0242	0.0046	0	0.0006	0.0169
C_8-C_9	0.15	0.12	0.0324	0.0087	0.0006	0	0.0111
C_{10+}	0.15	0.12	0.0779	0.0384	0.0169	0.0111	0

References

1. Coats, K. *An equation of state compositional model spe8284* (1979). Https://doi.org/10.2118/8284-PA.

2. Peng, D.-Y. & Robinson, D. B. A new two-constant equation of state. *Industrial & Engineering Chemistry Fundamentals* **15** (1), 59–64 (1976). Https://doi.org/10.1021/i160057a011 .

3. Firoozabadi, A. *Thermodynamics and applications of hydrocarbon energy production* (McGraw Hill Professional, 2015).

4. Lie, K.-A. *An introduction to reservoir simulation using MATLAB/GNU Octave: User guide for the MATLAB Reservoir Simulation Toolbox (MRST)* (Cambridge University Press, 2019). Https://doi.org/10.1017/9781108591416.

5. Møyner, O., Tchelepi, H. *et al.* *A multiscale restriction-smoothed basis method for compositional models* (Society of Petroleum Engineers, 2017). Https://doi.org/10.2118/182679-MS.

6. Yu, W., Lashgari, H. R., Wu, K. & Sepehrnoori, K. Co2 injection for enhanced oil recovery in bakken tight oil reservoirs. *Fuel* **159**, 354–363 (2015). Https://doi.org/10.1016/j.fuel.2015.06.092 .
